# Analytical Platforms for the Determination of Phospholipid Turnover in Breast Cancer Tissue: Role of Phospholipase Activity in Breast Cancer Development

**DOI:** 10.3390/metabo11010032

**Published:** 2021-01-04

**Authors:** Rosa Perestrelo, Marijana Petkovic, Catarina Luís Silva

**Affiliations:** 1 CQM—Centro de Química da Madeira, Universidade da Madeira, Campus Universitário da Penteada, 9020-105 Funchal, Portugal; rmp@staff.uma.pt (R.P.); marijana.petkovic@staff.uma.pt or; 2 Faculdade de Ciências da Vida, Unidade de Ciências Médicas, Universidade da Madeira, Campus Universitário da Penteada, 9020-105 Funchal, Portugal

**Keywords:** breast cancer, lipids, analytical platforms, statistical analysis

## Abstract

Altered lipid metabolism has been associated with the progression of various cancers, and aberrant expression of enzymes involved in the lipid metabolism has been detected in different stages of cancer. Breast cancer (BC) is one of the cancer types known to be associated with alterations in the lipid metabolism and overexpression of enzymes involved in this metabolism. It has been demonstrated that inhibition of the activity of certain enzymes, such as that of phospholipase A_2_ in BC cell lines sensitizes these cells and decreases the IC_50_ values for forthcoming therapy with traditional drugs, such as doxorubicin and tamoxifen. Moreover, other phospholipases, such as phospholipase C and D, are involved in intracellular signal transduction, which emphasizes their importance in cancer development. Finally, BC is assumed to be dependent on the diet and the composition of lipids in nutrients. Despite their importance, analytical approaches that can associate the activity of phospholipases with changes in the lipid composition and distribution in cancer tissues are not yet standardized. In this review, an overview of various analytical platforms that are applied on the study of lipids and phospholipase activity in BC tissues will be given, as well as their association with cancer diagnosis and tumor progression. The methods that are applied to tissues obtained from the BC patients will be emphasized and critically evaluated, regarding their applicability in oncology.

## 1. Lipids Structure and Metabolism

**Figure 1:**
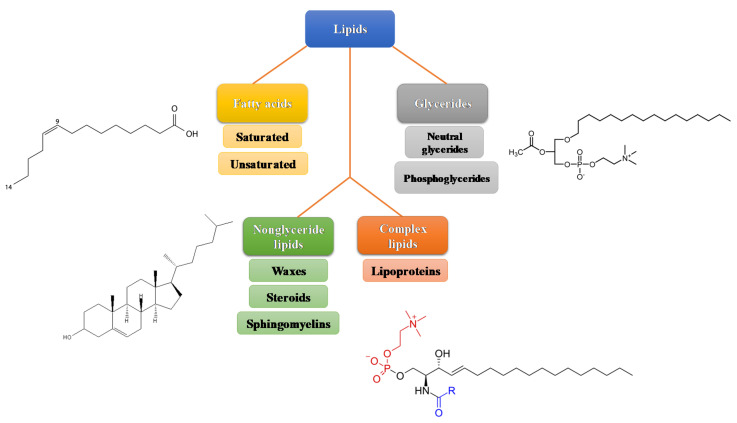
Classification of lipids and basic structure, adapted from [3,6].

Lipids comprise a family of molecules that are involved in the structural components of cell membranes, serving as an energy storage source, and in many signaling pathways [1]. They can be divided into several chemical families (e.g., fatty acids (FAs), phospholipids (PL)) as presented in Figure 1, their solubility in organic non-polar solvents being the common property for their classification. Cholesterol and FAs constitute the most representative molecules regarding their metabolic and nutritional functions. FAs are composed of carbon and hydrogen atoms, being linked by covalent bonds between carbons that can be single (saturated bond) or double (unsaturated bond), ranging from one to six double bonds. Regarding the FAs classification, FAs with no double bonds are called saturated fatty acids (SAFA), whereas FAs with one or more double bonds are named unsaturated FAs. Moreover, a molecule with one double bond is called monounsaturated fatty acid (MUFA), while those having two to six double bonds are so-called polyunsaturated fatty acids (PUFAs) [2].

In addition, lipids can take part in several physiological functions depending on their localization inside or outside the cell, and based on their chemical structure [3]. Lipids have four basic functions in living systems, more specifically, components of biological membranes, energy suppliers for cellular viability, modifiers to anchor certain proteins to the membrane, and as signaling molecules [1,3,4]. Due to their multiple roles, they are interesting candidates for the monitoring of the metabolic state of the organism, namely in the identification of characteristic profiles for many disease states, such as cancer [4]. The lipid metabolism comprises the oxidation of fatty acids (endogenous) for energy generation and the synthesis of lipids for degradation or transformation (catabolism) into several lipid-containing structures in the body when metabolized by enzymes [5].

The metabolism initiates in the intestine where the ingested triglycerides (TGs) are transformed in free FAs and a monoglyceride molecule by pancreatic lipases. In addition, this metabolism is associated with the one of carbohydrates, as the products of glucose (e.g., acetyl CoA) can be converted into lipids [3]. This step involves the conversion of nutrient-derived carbons into FAs. The biosynthesis of FAs and cholesterol is constrained to certain tissues including the one in the liver, the adipose tissue, the lactating breast, and in cancer tissues; there is also reactivation of the lipid biosynthesis [7]. Regarding inflammation, eicosanoids, including prostaglandins and leukotrienes as products of arachidonic acid metabolism are generated by enzymatic systems initiated by cyclooxygenases (e.g., COX 1 and 2), lipoxygenases (e.g., 5-LOX, 12-LOX, 15-LOXa, 15-LOXb), and the cytochrome P450 family [8]. Moreover, the overexpression of FAs and cholesterol biosynthesis, and also the utilization of free FAs from TGs, may lead to an increase in the levels of lipids with a signaling function which can contribute to different aspects of carcinogenesis [6,9,10], namely the eicosanoids and their involvement in the inflammation process, the reprogramming of fatty acid metabolism [11]. The metabolism of lipids is regulated by a network of signaling pathways that are interconnected and once there are perturbations, the entire lipid metabolic network will be compromised [5].

The research on lipid metabolism and signaling molecules during adipose tissue inflammation can be useful to understand certain diseases. This, combined with numerous analytical/statistical tools, will be described throughout this review.

## 2. Role of Lipids and Alterations in Breast Cancer

Being constituents of cell membranes and taking part in cellular functions, like survival, proliferation, and death, lipids are involved in chemical-energy storage, cell–cell interactions and cellular signaling and cellular membranes in tissues. These cellular processes are linked to carcinogenesis pathways, mainly to transformation, progression, and metastasis [12]. These biological functions of lipids make them putative biomarkers in the establishment of the metabolic state of the organism [4], and for the monitoring of disease progression [13].

One of the hallmarks of cancer is the disturbance of lipid metabolism, a complex physiological process, involving lipid intake, synthesis, and transportation throughout the organism [14]. Cancer cells have an extra demand for energy supply inducing alterations in lipid metabolism in order to allow the survival of these cells, namely by regulation of proliferation, differentiation, apoptosis, inflammation, and membrane homeostasis [10,15]. Several upregulated pathways are involved in cancer tissues, namely the de novo synthesis of FAs, including protein palmitoylation, formation of lipid rafts (lipid microdomains rich in glycoproteins and receptors), increased resistance to chemotherapy, regulation of redox balance, among others. This will lead to different cellular processes [16], namely hypoxia and limitation of nutrients supply. In addition, to overcome these conditions, cancer cells adapt their metabolisms, including that of the lipids [6]. The lipids mostly associated with BC comprise lysophosphatidic acid (LPA), which is known to play a critical role in the BC metastasis to the bone; glycosphingolipids and eicosanoids, including prostaglandins and leukotrienes, that have been implicated in several pathological processes, such as inflammation and cancer [9,10,17]. Furthermore, in healthy tissues, these pathways are regulated only by nutrition while in tumor tissues, they are dysregulated. Cancer growth, progression, and metastasis are achieved via the production of cytokines. In fact, Ju-Suk Nam et al. [18] showed that IL-8 and IL-11 were associated with bone metastasis in BC. In addition, LPA also induces the expression of osteolytic cytokines (IL-8 and IL-11) in BC cells by different LPA receptors. The overexpression of IL-8 by LPA may be through several pathways, namely the phosphatidylinositol-3-kinase (PI3K), and the nuclear factor kappa-light-chain-enhancer of activated B cells (NF-κB), whereas the protein kinase C delta type (PKCδ) pathway is responsible for the enhanced expression of IL-11. Furthermore, the secretion of IL-8 and IL-11 is mediated trough LPA in BC cells [4,18]. In addition, Baumann et al. [10] showed the importance of FA pathways in BC, namely in the human epidermal growth factor receptor 2 (HER2+) positive when compared with other BC subtypes. Clear differences in the lipidome of BC tissue compared to healthy counterparts have been found. Cífková and co-workers have discovered the upregulation of PLs with a high (4 double bonds) and a low degree (1 double bond) of unsaturation in tumor tissues, but also differences in the PC and PE content in various BC cell lines [19]. The differences in lipid content, fatty acid, and lipid metabolism are also evidenced within an individual BC subtype, based on mRNA arrays, as overviewed in the work of Monaco [20]. In addition, HER2 has been shown to upregulate the enzyme fatty acid synthase (FASN) transcription increasing the *de novo* FA synthesis. Furthermore, a study performed by Kourtidis et al. [21] showed that the levels of stored lipids were increased in HER2+ positive cell lines when compared with normal cell lines and that the existing free palmitate was cytotoxic to cells.

## 3. Phospholipases—Types and Involvement in Signaling Pathways

Phospholipases are key enzymes in the PL metabolism, and their aberrant expression and altered activity correlate with the development and progression of numerous diseases, including cancer. Apart from their involvement in the PL metabolism, these enzymes are in charge of the production of lipid second messengers that activate other proteins, which play a central role in the cellular processes such as cell growth, cell proliferation, and differentiation [22]. Keeping that in mind, it is not surprising that phospholipases are considered as target molecules and biomarkers for various types of cancers. There are, however, other enzymes that are involved in the lipid metabolism and the generation of second messengers, such as lysophosphatidylcholine acyltransferase 1 (LPCAT1) [23] or phosphatidylinositol phosphokinases [24], which are also important in the regulation of cellular processes, but they are beyond the topic of the current review. 

**Figure 2:**
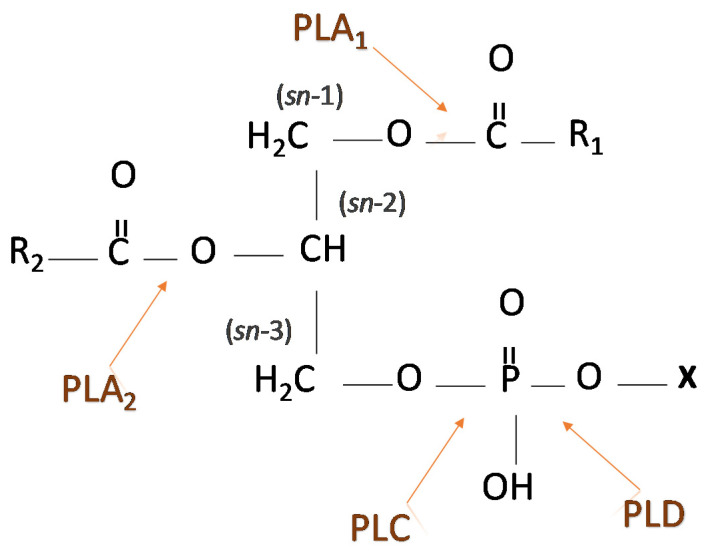
Position of the action of phospholipases. Abbreviations: PLA—phospholipase A; PLC—phospholipase C; PLD—phospholipase D; X—head group; R—fatty acid residue; sn—stereospecific numbering (position of C atom in the glycerol backbone). Phospholipids differ in their fatty acid composition and in the structure of their head group.

**Figure 3:**
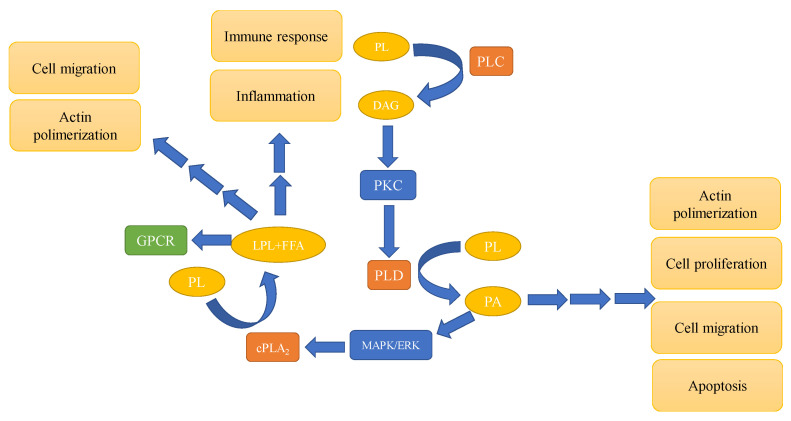
Simplified scheme of the interaction of phospholipases or products of their interaction with other enzymes in the intracellular signaling pathways and the processes they regulate. Abbreviations: cPLA_2_—cytosol phospholipase A_2_; PLC—phospholipase C; PLD—phospholipase D; PL—phospholipid; FFA—free fatty acid; DAG—diacylglycerol; PA—phosphatidic acid; PKC—protein kinase C; MAPK/ERK—mitogen activated protein kinase/extracellular-signal-regulated kinase; GPCR—heterotrimeric G-protein coupled receptor. Phospholipases exhibit their activity on the membrane, where their substrates, PLs are located. Multiple arrows in the figure indicate the effect with several intermediate enzymes, whereas one arrow indicates a direct interaction of the lipid second messenger and the enzyme.

Phospholipases are divided into several chemical families depending on the substrate and position of action (Figure 2), but each family has also several subtypes and isoforms that are in most cases differently regulated. For instance, phospholipases can be activated by phosphorylation by protein tyrosine kinases, by the increased concentration of intracellular calcium, or by any other downstream processes after binding of the activator to the corresponding receptor on the surface (Cf further in the text). Since their substrates, PLs, are in the plasma membrane, phospholipases exhibit their action only when they are associated with the membrane. Some of them, such as secretory phospholipase A_2_ (sPLA_2_) can be excreted from the cell. Finally, products of the phospholipases activity can remain in the membrane, they can be released from it thus reaching the target molecule, or they also can be further metabolized. The summary of their activity, position in the signaling pathway, and potential targets are presented in Figure 3. 

Phospholipases A (PLA) catalyze the cleavage of fatty acids from the PL, releasing FFA and leaving the corresponding lysophospholipid (LPL) in the membrane. Depending on the position from which the FFA is released, there are two groups of PLA: namely phospholipase A (PLA_1_) that catalyzes the hydrolysis of FFA from the *sn*-1 position of PL, and PLA_2_, that catalyzes the hydrolysis of FFA from the *sn*-2 position in the PL. PLA_2_ comprises 5 subtypes that differ in their mass, localization, substrate specificity and regulation of activity. These are cytosolic PLA_2_ (cPLA_2_), which is activated by phosphorylation, secretory PLA_2_ (sPLA_2_) whose activity is associated with high concentrations of Ca^2+^ and acts mostly extracellularly, calcium-independent (iPLA_2_), lysosomal PLA_2_,) and platelet-activating factor acetylhydrolase (PAF-AH) (review in [25]). Each of them is involved in the regulation of important cellular functions, and their up-or down-regulation is postulated to be associated with the progression of the disease. This is the reason why this group of enzymes are considered as target molecules in the anti-tumor therapy [26]. The signaling role of PLA_2_ and the products of its activity is demonstrated in cancer cell lines, in which the inhibition of the PLA_2_ activity increases the sensitivity towards chemotherapy of breast and cervical epithelial cell line Ect/E6E7 [27]. 

Cytosolic PLA_2_ and iPLA_2_ are ubiquitous enzymes located in the cytosol, but upon activation, translocate to the plasma membrane. Binding of a spectrum of molecules, such as cytokines, tumor necrosis factor α(TNF α), hormones, epidermal growth factor, and others to the cell leads to the activation of these enzymes [28], and as a result of their activity, several intracellular processes are triggered. CPLA_2_ and iPLA_2_ might use similar substrates where the regulation mechanism of their activity is the phosphorylation through the MAPK/ERK kinase pathways. These pathways activate cPLA_2_ [29], where its active form is the oligomerization [30]. Both enzymes bind to phosphatidylinositol 4,5-bisphosphate (PIP_2_) and/or PC and catalyze the release of mostly arachidonic acid from the *sn*-2 position, leaving the corresponding lysophospholipid in the membrane [31]. FFA are further used as substrates for an enzyme involved in the regulation of inflammation [32], whereas the LPC is usually considered to be an intermediate in the PL remodeling [33]. However, there is some evidence that this lysolipid can also act as a signaling molecule, and activate protein kinase [34,35]. In contrast to intracellular types of PLA_2_ that are involved in the intracellular signaling, the secretory enzyme, sPLA_2_, is secreted from the cells, and exhibits the activity extracellularly, either on the outer side of the plasma membrane or on extracellular lipids, by generating intercellular mediators, lyso-lipids, and FFA [36]. 

Another phospholipase, PLC, catalyzes the cleavage of the phospho-head group of PLs (Figure 2), yielding and increased concentrations of diacylglycerol (DAG) in the membrane, and IP_3_, which interacts with the corresponding receptors and releases Ca^2+^ from the endoplasmatic reticulum [37]. DAGs with Ca^2+^ activates protein kinase C (PKC), which is one of the central enzymes in the regulation of the cell cycle [38]. Various stimuli that activate G-protein related pathways result in the activation of several PLC isozymes, and the small G-proteins from the Ras, Rac, and Rho family can also be activators of this family of phospholipases [37]. Tyrosine phosphorylation and phosphatidic acid (PA), which is generated by the activity of PLD (described later), can directly activate PLC [39], further affecting the heterotrimeric G-protein downstream intracellular events. Upon increased activity of PLC and higher content of DAGs in the membrane, PKC is transported to the membrane, where it interacts with DAGs, and phosphorylates numerous substrates. Furthermore, an increased concentration of phosphatidylserine (PS) and Ca^2+^ are required for the PKC activity [40]. PKC phosphorylates transcription factors, receptors, and is involved in the regulation of immune response [41], where this protein kinase and PLC are important enzymes in the control of the cellular cycle. Therefore, an aberrant PLC activity can contribute to tumorigenesis [42]. 

There are two isozymes of PLD in mammalian cells, and both catalyze the cleavage of the PL head group resulting in increasing amounts of PA. This enzyme, like other phospholipases, participates in membrane remodeling, but PA is also the well-known second messenger that is involved in processes like endocytosis, degranulation, cell cycle progression [43], and regulation of NADPH activity in human polymorphonuclear leukocytes [44]. Increased expression of PLD, its altered activity and mislocalization, are all postulated to be involved in cancer development. The processes that are regulated by PLD and the product of its activity, PA, are linked to tumorigenesis of several cancers, including breast, prostate, colon, and other types (reviewed in [45]). Inhibitors of PLD are also shown to suppress the growth of the patient-derived prostate cancer cell line [46] and PLD activity and production of PA are associated with the mechanism of invasion of BC cells in a xenograft model [47]. The most probable mechanism is through the interaction of PA with actin, which is an integral part of the cytoskeleton affecting its polymerization. The aberrant activity of phospholipases is associated with tumor initiation, development, progression, and metastatic potential. The complexity of intracellular regulatory mechanisms and the inter-connection between numerous actors in the signaling pathways emphasize the need for complex data analysis and, thus, a combination of experimental and statistical tools.

## 4. Breast Cancer Lipidomics—In Tissue Approach

### 4.1. Lipid Extraction Procedures

Independently of the analytical platform used for lipidomics, the sample preparation technique is of crucial importance. The procedures are mostly based on the lipid extraction from the homogenized tissues, which were previously frozen and stored at a low temperature (−80 °C), to prevent the activity of enzymes involved in the lipid metabolism. Excellent review of advanced methods of sample preparation technique for lipidomics is published by Teo and co-workers [48], and Aldana et al. [49]. Authors emphasize that basically, when dealing with tissues, all necessary steps must be undertaken to prevent any changes in the lipid composition upon handling. The selection of the extraction method depends on the target subset of lipids, e.g., their polarity and concentration [50]. The most challenging aspect off the extraction is the untargeted lipidomics, because of a high variety of lipid structures and variable polarity, which might lead to low recovery of certain species in one solvent system. The most commonly applied solvent systems are chloroform/methanol mixtures (so called, Bligh and Dyer or Folch methods) with various water moiety or the addition of low concentrations of HCl or NaCl to increase polarity [51,52]. If the quantification of lipid species is of interest, then the internal standard (the same lipid species, usually labeled with stable isotopes) is added to the sample. It is used as the quality control (QC) for the sample recovery from the biological matrix (reviewed in [53]). Concerning the potential susceptibility of individual component to suffer hydrolysis in an acidic environment, caution is needed when handling these samples. In all cases, to avoid the oxidative damage of lipids, they should be stored in an inert atmosphere and dissolved in a solvent compatible with the analytical platform that will be applied for lipidomics. Although for the liquid (LC) and gas chromatography (GC) coupled with mass spectrometry (MS), lipids must be extracted and solvents changed according to their compatibility with the method, for imaging techniques (discussed in Section 4.3), tissue slices (dried, formalin-fixed or frozen) must be prepared and no previous separation is required. This is, however, the advantage over the platforms that require lipid extraction. On the other hand, there is a question of detectability of individual species in the complex milieu. 

MS coupling with chromatographic separations or direct infusion mass spectrometry (DIMS) is an approach extensively used in lipidomics research, with potential applicability for high-throughput clinical analysis for identification of putative cancer biomarkers and early diagnosis screening [4,54].

### 4.2. Chromatographic Separation Coupled with Mass Spectrometry

The chromatographic separation, such as LC and GC, prior to MS provides numerous benefits like separation and detection of isomers and isobars, reduced ion-suppression effects, chance to separate the target analytes based on their physicochemical properties (e.g., polarity, vapor pressure, chargeability), and a substantial amount of information for the complex samples [4,12,55,56]. The efficiency, resolution, and retention time of separation depends on the lipids structure and stationary phase used [4]. This is one of the most frequently used platforms in lipidome/metabolome research that can be also applied for quantification and structural elucidation of lipids. 

Gas chromatography coupled to mass spectrometry (GC-MS) is used for the identification and quantification of neutral lipids (e.g., triglycerides, cholesteryl esters). Budczie et al. [57] analyzed a cohort of 271 BC and 98 normal tissues using GC followed by time-of-flight mass spectrometry (TOFMS) and reported 13 tumor markers for discrimination between BC and normal tissue with sensitivity and specificity of roughly 80%. 

On the other hand, LC-MS is a powerful analytical platform for the separation of polar lipids, namely phospholipids (PLs) and their subclasses [12]. Several software packages enable automated lipidomics and identification of individual lipid species based on the fragmentation of individual peaks after their chromatographic separation, i.e., LC-MS/MS (reviewed in [58]). The liquid chromatography—electrospray ionization mass spectrometry (LC–ESI/MS) platform can improve sensitivity and accuracy for low abundance lipids, being normally used to targeted and untargeted lipidomics. Nowadays, noteworthy developments in chromatographic resolution, reduction of ion suppression and time of analysis are attained using ultra-high performance liquid chromatography (UHPLC) and ultra-high performance supercritical fluid chromatography (UHPSFC), due to the use of columns with sub-2 µm particles and higher operational pressure [4,12]. UHPSFC is the most used for lipids separation since it showed applicability for non-polar and polar lipids in short analysis times [54], whereas hydrophilic interaction chromatography (HILIC) and normal phase liquid chromatography (NPLC) is used to separate polar and non-polar lipids, respectively [4]. NPLC show lower reproducibility compared to HILIC and is less compatible with MS as the applied mobile-phases are greatly volatile and have low ionization ability [56]. In this sense, Cífková et al. [59] proposed HILIC-HPLC/ESI-MS to establish differences in lipidomic profile between human BC and surrounding normal tissues. The results showed that the phosphatidylinositol (PI) provides the greatest difference between concentrations of normal and tumor tissues (more than four times) for the analyzed patients, whereas PE species (P-36:4, P-38:5/O-38:6, and P-38:4/O-38:5) were more abundant in normal tissues. Vosse et al. [54] developed an extended phospholipid profiling of a cell culture model of conditional oncogene overexpression in MCF-7/NeuT BC cells using HILIC-HPLC/ESI-MS. The comparison of control and oncogene-induced MCF-7/NeuT BC cells displayed changes in bis(monoacylglycero)phosphate (BMP) species distribution. Zhang et al. [60] analyzed tissue samples of patients with BC using derivatization through the Paternò-Büchi reaction with liquid chromatography tandem mass spectrometry (LC–PB–MS/MS). A total of 143 unsaturated PEs (71) and PCs (72) molecular species were identified at C=C location level, but only 6 lipid species (PC 32:1, PC 34:2, PC 34:1, PE 36:1, PE 38:4, and PE 40:7) showed significant differences. The ratios of C=C isomers may be used for the discovery of lipid biomarkers, as the position of the double bond (e.g., C18:1, the ratio between isomer Δ9 and Δ11 in PEs and PCs) within the individual PLs in BC tissue, is more related to the morphological changes of breast cancer, and demonstrates less interpersonal variability, thus being a reliable biomarker for disease progression. Hilvo et al. [61] conducted comprehensive lipidomics in 267 human BC tissues using UHPLC-MS by using LC-MS/MS platform and the univariate/multivariate analysis. The data extraction was done by targeted lipidome, using the Metaboanalyst 4.0 Package^®^ (CA, USA) [62] by setting the criteria for individual species to be present in more than 80% of the samples giving the high intensity in the spectra and low variations in the QC measurements. This study is one of the rare comprehensive lipidomics study using extracts from the BC tissues. 

The results showed that products of de novo FA synthesis incorporated into membrane PLs, such as palmitate-containing PCs, were increased in tumors as compared with healthy breast tissues. Thus, PLs may have diagnostic potential as well as, modulation of their metabolism may provide therapeutic opportunities in BC. For UHPSFC, supercritical carbon dioxide (CO_2_) with modifiers (e.g., acetonitrile, methanol, propanol) is the most common mobile phase used, with the purpose of controlling solvation, elution strength and polarity [4]. Thus, the benefits of UHPSFC are based on higher diffusion coefficients and lower viscosities compared to LC. Similar stationary phases as in UHPLC have been used for lipidomics profile in UHPSFC [54].

### 4.3. Direct Infusion Mass Spectrometry (DIMS)

DIMS is an analytical platform without prior chromatographic separation of lipids, which requires less time, and low sample volume, has high sensitivity and is more reproducible than other platforms. Nevertheless, DIMS has the disadvantage of ion suppression [12]. Ion suppressions occur when easily ionizable species are abundant in the mixture, such as choline-containing PLs [63]. This may cause underestimation of other components in the mixture, but some approaches can help to solve this problem. The easiest approach is the acquisition of the same spectra mixture in the negative ion mode, or the addition of ions, such as Cs^+^ or NH_4_^+^ to the mixture to facilitate ionization and shift the masses towards a higher region and discover overlapping signals [64]. The MS instruments are composed of three modules: an ion source (transforms solid, liquid or gaseous molecules into ions), a mass analyzer (sorts the ions by their *m/z* using acceleration or deflection) and a detector (counts existing ions and deliver a mass spectrum after computer determination) [65]. Electrospray ionization (ESI) and matrix-assisted laser desorption/ionization (MALDI) are the most common soft ionization techniques used, and they can be combined with all types of MS analyzers, like time of flight (TOF), orbital ion trap and hybrid instruments (e.g., quadruple/TOF) [56,65]. 

MALDI-TOF-MS appears to be the most optimal technique for lipidome research [65], due to its easy operation and requirement of inexpensive matrixes for sample preparation. Furthermore, a screening of a large set of samples can be carried out in a short analysis time in a fully automated instrument, due to advanced laser technology and hardware [66]. The selection of the matrix for a specific target is the crucial step in MALDI-TOF-MS analysis. A suitable matrix should permit the generation of homogenous co-crystals with the analyte targets and should be stable under high vacuum to avoid its sublimation. Moreover, the matrix should present a high absorbance at the emission wavelength of the laser, high sensitivity (excellent signal/noise ratio), a low tendency of analyte–matrix ion cluster formation, as well as a low self-background. 2,5-Dihydroxybenzoic acid (DHB) is the most common matrix used in lipidomics research, and can be applied to both positive and negative ion mode [65]. After ionization, the ions are quickly accelerated though a strong electric field in the ion source, and then the ions enter the TOF tube or drift region, permitting separation according to the velocity, and consequently *m/z* ratio [67].

Recently, Silva et al. [68] established a lipid biosignature of BC tissues using MALDI-TOF-MS, and significant differences (*p* < 0.05) on the ratios of LPC 16:0/PC 16:0_18:2 between active carcinoma tissues and cancer-free tissues, as well as for BC stages II and III, were observed. Cho et al. [69] proposed a fine needle aspiration (FNA) followed by MALDI-TOF-MS to characterize lipid biomarkers for diagnosing accuracy of BC, and the results indicated that PCs and TGs can be used as biomarkers for the diagnosis of BC. The results obtained with this approach were confirmed by MALDI-mass spectrometry imaging (MSI) analysis. In addition, Kang et al. [70] demonstrated the ability of MALDI-MS in profiling lipids to classify human BC samples according to the intrinsic subtype. Although both ionization techniques provide much information, when applied directly, mostly structural and semi-quantitative information could be gathered.

### 4.4. Mass Spectrometry Imaging (MSI)

The MSI is the most frequently used analytical platform for in situ molecular analysis of cancerous cells and tissues with the purpose of recognizing tumor margins, and categorizing primary tumor tissues concerning their chemo-response and metastatic stage, as well as analyzing drug response and resistance [67]. In this analytical platform, a tissue section covered with a matrix is located in the ion source and spectra are acquired by shooting sequential parts of the tissue surface [65]. Through the use of MSI, it is possible to gather quantitative and structural information, but also the spatial distribution of lipid species in the tissue.

For lipid imaging, several desorption ionization techniques have been proposed, with MALDI and desorption electrospray ionization (DESI) being the most applied [4]. An advantage of DESI for the analysis of lipids and small molecules is that no sample preparation is required, whereas MALDI requires the matrix deposition [67]. Discriminatory lipid signatures between cancerous and normal BC have been identified using DESI-MSI [71], with the delineation of tumor margins possible through the analysis of PI (18:0/20:4).

MALDI-MSI combines the sensitivity and selectivity of MS with the spatial analysis provided by traditional histology, offering unbiased visualization of the spatial arrangement of biomolecules (e.g., peptides, proteins, lipids, glycans, drug metabolites) in tissue and cells [67,72]. Commercially offered MALDI-MSI operates in microprobe mode, with spatial resolution below 20 µm, in which a raster by the laser is carried out over a tissue area, producing mass spectra at each ablation point [72]. Data files sizes obtained by MALDI-IMS can be large with consequent low-resolution imaging even for routine use. Individual images can produce thousands of spectra, which requires high processing time, computer costs, and data storage [72]. In order to remove the background signals from matrix degradation observed in MALDI-MSI, the method nanoparticle-assisted laser desorption ionization (n-PALDI) was developed, which uses nanoparticles as matrixes/substrates for ionization [67].

Wang et al. [73] proposed MALDI-MSI to establish the lipidomic profile of two poorly invasive and two highly invasive BC cell lines to recognize the differentially accumulated lipids linked to the invasive phenotype. A total of 31 lipids were identified as upregulated and 8 lipids as downregulated in highly invasive BC lines compared to poorly invasive BC lines. Moreover, Ide et al. [74] used MALDI-MSI to visualize PCs and LPCs in human BC tissue, and three species of PCs were relatively abundant in cancerous when compared to the remaining sections. MALDI-IMS was used to determine the distribution of tamoxifen in both ER-positive and ER-negative BC tumor tissues [75]. On the other hand, Mao et al. [76] reported a novel air flow-assisted ionization (AFAI) coupled with MSI to be used in ambient environments in order to differentiate BC using lipidomics profile. The obtained results revealed that numerous subtypes and histological stages of IDC and DCIS can be discriminated using AFAI-MSI, as PLs were more predominant in breast invasive ductal carcinoma (IDC) than in breast ductal carcinoma in situ (DCIS), whereas FA were more abundant in DCIS than in IDC.

### 4.5. Batch Effects in Breast Cancer Lipidomics and Identification of Lipids

Lipid profiling and biomarker discovery start with the collection of a large number of samples and a high number of large data that are analyzed. There are always differences caused not only by intrinsic properties of BC lipids but most importantly by analytical methods, which involve a small drift in the mass of the same ion detected between different sets of samples (e.g., batches). Since this is unavoidable in automated analyzes, analytical scientists have developed approaches to overcome this problem and to increase the accuracy, precision, and reproducibility of measurements. This approach can be used in LC-MS as well as in DIMS platforms.

This problem can be solved by introducing the isotopically labeled standards as the QC samples and it has been successfully applied for untargeted lipidomics of blood samples from patients suffering of Alzheimer’s disease [77], as well as for the lipidomics of breast cancer cell lines [78]. In the latter case, the differences in the lipid composition between various BC cell lines were discovered and quantified by LC-MS platform and statistical analysis. QC-derived ions with the smallest RSD of mass drift in the spectra, both positive and negative, are chosen as standards for the signal normalization, the correction factor both for the signal position and the intensity/concentration ratio. By this approach, the up- and/or down-regulated lipids in the BC samples were revealed [19]. Careful selection of the internal standards/QC samples should be made, as they must correspond to the lipid class of interest and its physical properties in terms of ionization and fragmentation pattern [79].

QC standards are not only useful for purposes of quantification, but also for a reliable identification of lipid species, achieved by fragmentation. In tandem mass spectrometry, (MS/MS) fragmentation is induced by collision with a gas, usually He or Ar. Fragmentation of ions is necessary due to a high variety of lipids that are present in the tissues, and can be classified into several categories. Each category yields a characteristic fragment that can be assigned to the lipid class, and different fatty acids can also be identified. Table 1 presents the most common classes of phospholipids that can be identified in MALDI. As for example by using HILIC ESI MS/MS, Cífková and co-workers [19] have been identified upregulated PL species in BC tissue compared to a healthy one, but also detected downregulation of PLs with polyunsaturated fatty acids, in particular those with 3 or 4 double bonds. An overview of lipids generated fragmentation ions if given in a work of Hutchins and co-workers [80].

#### Detection of Oxidatively Modified Lipids in Breast Cancer Tissue

Apart from the confirmation of their signal identity, the oxidative modifications of lipids can also be discovered by fragmentation. The most characteristic oxidatively modified fragments are those that arise from peroxidation of the double bond of fatty acids’ residues [83]. Breast cancer cells contain higher content of polyunsaturated fatty acids [19,84], which are more prone to oxidation. Thus, it is expected that more lipid-oxidation products will be detected by BC lipidomics, upon the response of immune cells, such as neutrophils and macrophages that are involved in the removal of tumor tissue (reviewed in [85]). However, it seems that the concentration of products of oxidative stress decrease with the BC progression [86]. Detection of oxidatively modified PLs by MS can be easily achieved by applying “soft ionization techniques”. However, most studies have been performed with model systems, such as PL mixture/vesicles treated with increasing concentrations of H_2_O_2_ or HOCl (overview in [87]. Modification of PLs, such as chlorohydrin (addition of Cl to the double bonds) or aldehydes were detected at lower *m/z* ratios. If PLs with highly unsaturated fatty acids are subjected to oxidative modification, lyso-phospholipids are detectable in the MALDI TOF mass spectra [88]. These lyso-lipids are also products of the PLA_2_ activity (Cf. Chapter 3, and [25]). Therefore, although oxidative stress has a role in BC development and malignant potential, there are no publications dealing with the detection of oxidatively modified lipids (oxidative lipidomics) by MS-based approaches in BC tissues. One of the rare reports is concerning the expression of aquaporin and the differences in lipid profiles in BC cells upon oxidative stress [89]. However, the study focuses on the analysis of fatty acids, and GC was used for their analyses. Differences in the fatty acid content and composition were found between BC cell lines of various malignancies and hormone responsiveness. The lowest content of saturated FA was found in estrogen-responsive cell line, whereas the HER positive cell line has the highest content of peroxidation products, without any treatment. The later finding correlates positively with the content of monounsaturated FA. The products of lipid oxidation can be reactive. Malondialdehyde (MDA) is generated by peroxidation of polyunsaturated fatty acids, and its accumulation in the membrane might lead to changes in membrane fluidity. Another product, 4-hydroxy-2-nonenal (HNE), or PUFAs with six double bonds, is highly reactive and binds to proteins leading to changes in protein structure in cancer cells. An increase in the content of lipid peroxidation markers in cancer cells results in their sensitization towards therapy with other anti-cancer agents [90]. Unsaturated fatty acids and products of their oxidative modification play a role as immunomodulators in tumor progression. A similar role is assigned to oxysterol species (oxidative modification of cholesterol) that can be a mitogenic factor in the estrogen receptor positive BC [91], and it has been demonstrated that they can have a pro-metastatic role in BC [92].

The level of oxidative stress increases upon chemotherapy and thus; it is important to monitor the products of lipid peroxidation by performing oxidative lipidomics. Despite an increased sensitivity and resolution of MS, there are no applications to BC tissue in oxidative lipidomics.

As a complementary technique, the MS-imaging can replace the need for lipid extraction and potential modification during the procedure. However, as was discussed, this approach also has some limitations [93], which can be overcome by a careful choice of the preparation technique, addition of a QC standard, and the selection/concentration of the matrix.

### 4.6. Statistical Analysis

Statistical analysis is a crucial step for clinical analysis and biomarker discovery. One of the main advantages of lipidomics is the potential for assessing several classes of lipids simultaneously. On the other hand, the pool of data obtained can represent a problem for statistical analysis, since the majority of these statistical tests are univariate, which requires more replicates than the number of variables. If the data obtained adopted a normal distribution, the Student’s t-test (for two group comparisons) and analysis of variance (ANOVA, for multiple group comparisons) are the most used statistical tests. However, if the data obtained does not adopt a normal distribution, another statistical tool should be used, like the Kruskal–Wallis test. In addition, in cases that have more variables than samples, these univariate tests can be used in combination with corrections for multiple testing [94].

In this sense, multivariate statistical tests (e.g., principal components analysis (PCA), partial least squares discriminate analysis (PLS-DA)) are the most commonly used for lipidomics research, because it is necessary to handle a large number of variables and visualize these datasets [94]. The multivariate statistical tests are extensively applied in exploratory studies to obtain dataset pattern recognition through the relationships between groups and can be organized into two sub-groups: unsupervised and supervised approaches [95]. In exploratory studies, unsupervised methods (e.g., PCA) are the most used since the modeling process is based only on explanatory variables, without external interference of the user [96]. PCA showed a projection of dataset into low dimensional dataset according to orthogonal transformation, that converts the variables from a set of observations into score vectors and loadings, named principal components [95]. For this reason, an unsupervised approach is a preferential option for the initial visualization of the dataset, consequently allowing for the identification of outliers and the determination of what are the major effects measured in a research [94]. On the other hand, supervised approaches (e.g., PLS-DA) are more suitable after the explorative studies and the variable selection are carried out, since that the following step is the processing of dataset to develop a predictive response model to classify new samples (e.g., diagnostic tools), identify valuable variables (e.g., biomarkers) and/or explore the mechanism pathways (e.g., lipid pathways) [95].

Finally, it is crucial to validate the predictive model in order to check its performance in appropriately predicting the hypothesized relationships between variables and response [95]. For this purpose, a cross-validation (CV) method is the most used, since it provides a qualitative and quantitative analysis of the model ability to predict new independent samples without collecting additional data. In this method, the data are split into two groups. One group is used to develop a predictive model employing the values of continuous and predictor variables (training group), and the other group is used to evaluate the performance of the predictive model (validation set) [95]. The most common CV test is the K-fold, which is based on a random partition of the original dataset into equal-sized subsamples (k); more specifically, the K-CV leave-one-out cross-validation (LOOCV) and the Monte Carlo cross-validation (MCCV), the former being used in small datasets [97]. Kang et al. [70] discriminated BC cancer from normal tissue with the prediction accuracy of 94% (*p* < 0.01). Luminal, HER2+, and triple-negative tumors showed different lipid profiles, as demonstrated by permutation for 0.632 bootstrap cross-validated misclassification rates. Mao et al. [76] evaluated the predictive ability of the DCIS versus the IDC model in performing an external test using 10 BC samples from 5 specimens of DCIS and 5 specimens of IDC. The classification of specimens in the subtype and grade validation sets displayed 100 and 79% agreement with the histopathological diagnosis, respectively. Cho et al. [69] used a receiver operating characteristic (ROC) curve to process the data collected from the FNA sampling and the MALDI-TOF-MS. The area under the curves (AUC) ranged from 0.832 to 0.919, which was revealed to be strongly associated with sensitivity and specificity. In addition, the ROC results were in agreement with PCA, meaning that PCs and TGs can be putative biomarkers for BC diagnosis. Another study obtained significant differences (*p* < 0.05) in lipid levels between BC and normal tissues using tissue spray mass spectrometry, for PI, PCs, and SM [98]. Moreover, the results obtained by orthogonal partial least-squares discriminant analysis (OPLS-DA) classification of the tissue revealed 100% sensitivity and specificity when compared to histological analysis [98]. Finally, Cifková et al. [19] correlated the lipidomic profile of cell lines and tissues of BC patients using HILIC/ESI-MS combined with multivariate data analysis (e.g., PCA, OPLS-DA). The obtained results showed a clear differentiation between BC and normal tissues, PLs being the most upregulated lipids with a low degree of unsaturation, whereas the most downregulated lipids were PL containing polyunsaturated fatty acyls (e.g., 20:4), plasmalogens, and ether lipids.

## 5. Breast Cancer Lipidome: Comparison of Results Obtained with Cell Lines and Breast Cancer Tissues

Most information about the lipid metabolism of BC and potential biomarkers are obtained from the cell lines. Although well-established BC cell lines correspond to various BC types and grades, there are numerous gene mutations therein that cause significant differences between them and the primary culture. Unfortunately, sufficient amounts of BC tissues with various grades is difficult to obtain to perform statistical analysis and to draw conclusions about the up- or down-regulated lipid species.

By applying UPLC-QTOF-MS, Eriksson et al. revealed different lipidome profile between 7 BC cell lines, with the estrogen- and progesterone -receptor positive, showing a higher level of TGs and lower level of ether-PE [78]. Cell lines that are overexpressing the receptor for human endothelial factor, had a higher level of TGs, PCs, and PE with short chain fatty acids, but triple negative BC cell lines demonstrated an increased content of PCs. The work of Dória et al. [99] showed that the relative content of PE was highest in non-malignant BC cell lines, whereas the content of PA was highest in metastatic ones. Although there are the differences that can be assigned to the progression of BC, one should keep in mind that the results are obtained with the cell lines.

In the work of Cifková [19], significant differences were obtained in the content of individual PL species between the BC cell lines and healthy cells, but also between the BC tumor tissue and the healthy mammary counterpart. The differences were obtained in both the upregulated and down-regulated PL species. For instance, the level of PC (32:1) was significantly higher in the BC cell line compared to the healthy cell culture, whereas this difference was not detected in tumor tissue compared to the healthy counterpart. As described, the different lipidome profile was also obtained between BC cell lines and tumor tissues, emphasizing the importance of the object of study.

Based on the lipidome profile, the conclusions about enzyme activity could be drawn, but there are few studies deducing an enzyme activity based on lipidome results. For instance, an increased LPC/PC ratio in the extracts of BC tissues implies either an increasing PLA_2_ activity with the disease progression (Silva et al. [19]) or a decreased acyltransferase activity. These conclusions could not be drawn simply on the levels of expression of these enzymes, as this does not correlate directly with their activity.

## 6. Expression and Activity of PLA_2_ in Breast Cancer: What Is the Best Method to Determine It in the Tissue?

Although there are pieces of evidence that the products of the cPLA_2_ enzyme activity are involved in carcinogenesis, the role of cPLA_2_ in tumor progression and migration is not completely clear. It is assumed that AA could promote the BC migration through the activation of focal adhesion kinase (FAK), but the activation of cPLA_2_ by the cell stimulation with EGF also increases tumor migration and chemotaxis [100]. Additionally, LPA is a mitogenic factor [101], that is overproduced in ovarian cancer cells, in an iPLA_2_-dependent manner, and inhibition of this enzyme suppresses the proliferation of ovarian cancer [102]. sPLA_2_ has an important role in the progression of various cancers, including BC, and its aberrant expression was associated with BC malignancy [103]. It was also shown that the increased expression of sPLA_2_-II is closely correlated with the clinical staging, histological grading, and lymph node metastasis of breast infiltrating ductal carcinoma [104]. In these studies, the enzyme concentration was determined from tissue homogenates by radioimmune assay and immunohistochemistry, respectively. In addition to the overexpression of sPLA_2_ in the BC tissue, plasma and sPLA_2_ were also considered as the diagnostic markers for the BC, in particular in patients in later diseases stages [26].

Since the concentration of the PLA_2_-derived second messengers directly regulates the downstream events that are involved in the carcinogenesis/tumor progression, not only is the concentration of these enzymes important, but also their activity. This statement is supported by the findings in which the inhibition of PLA_2_ sensitizes cancer cells towards further chemotherapy [27,105]. Besides, our recent results strongly suggest the correlation of the PLA_2_ activity with cancer grade [68]. Now, the question that arises is how one can study the activity of PLA_2_ in the tumor tissues, without affecting it during the assays and the potential fractionation and/or purification. For such a complex system like tumor tissues, but also for samples obtained from the BC patients (e.g., urine, saliva), it is most likely that several approaches have to be combined involving statistical analysis of the results, in a way that the alterations in the PLA_2_ activity can be associated with the tumor grade.

Changes in the expression of PLA_2_ can be monitored by immunohistology and specific staining of PLA_2_-bound antibodies, which is a routine procedure performed for the assessment of PLA_2_ in prostate cancer [106], or in breast cancer [104]. On the other hand, PLA_2_ can also be isolated from BC tissues and its concentration determined by radioimmunoassay (RIA) [103].

The situation is somewhat more complex when there is an assessment of the enzymatic activity of PLA_2_. This is of high significance because the products of its activity are second messengers that can regulate the activity of other enzymes involved in tumor progression. For the investigation of enzyme activity, the isolated enzyme can be incubated with the substrate, mostly PC, which must be labelled fluorescently. Based on the changes in fluorescence intensity, the enzyme activity can be determined. Although some critical points could be raised against the application of a non-natural substrate, this approach was used in the study of BC biomarkers in plasma [26]. In case where natural substrates are used for the assessment of the PLA_2_ activity, lipid extraction and separation are usually applied, and the concentration of either substrate or products of the PLA_2_ activity (FFA or LPL) can be determined by LC-MS or MS only.

The advantages of the application of soft ionization mass spectrometry techniques, such as ESI or MALDI for determination of the PLA_2_ activity is that the relative concentrations of LPLs as the products of PLA_2_ activity can easily be determined by the procedure without labelling, and that it can be applied to natural substrates. This was done previously by MALDI and pancreatic PLA_2_ [107,108] and this approach was successfully used for the determination of kinetic parameters of the enzyme activity, as well as for the calculation of the binding constants for metallodrugs. Furthermore, a method for fast and sensitive determination of PLA_2_ activity by LC-ESI-MS was developed [109,110]. An additional advantage of both approaches is that it is possible to distinguish between various substrates, thus PLA_2_ does not need to be incubated by isolated PL, a complex PL mixture can be used, and substrate preferences identified. By using the MALDI-MS platform, it was possible to determine the activity of PLA_2_ in tissues. This approach comprises the extraction of lipids from tissues sections obtained from BC patients of different stages, and the determination of ratios between the various lipids-substrates calculated from MALDI TOF mass spectra [68]. However, the number of samples was too low in this study, and a larger cohort is required to get more significant results in both, with statistical significance, which will allow for further identification of BC biomarkers.

Compared to other analytical platforms, MSI is advantageous, because it provides information about the special distribution of molecules of interest, as well as the concentration, which makes it a valuable tool for biomarker discovery [111,112], as discussed in Chapter 4.3. This approach is advantageous because it does not require any purification or sample derivatization, and therefore it is superior in that sense when compared to other methods. After the spectra acquisition, it is possible to correlate tissue regions with overexpressed PLA_2_ and the products of its activity. Theoretically, MALDI-MSI can be used for evaluation of enzyme activity within the tissues. For instance, regions with increasing concentrations of LPC were identified in injured ischemic brain [113], and they could be correlated with histopathological images and regions with an overexpressed enzyme in cancer tissues. Changes in the PI concentrations and diversity of PI species were also discovered in prostate cancer tissues, and a different pattern was obtained in prostate cancer tissue and healthy surrounding epithelium [114,115].

Unlike MALDI-MS, MALDI-MSI can be treated statistically, making the identification of biomarkers even easier. Having that in mind, it is clear that MALDI-MSI is a valuable analytical platform in lipidomics [93], as well as in general metabolomics [116]. In many studies, thousands of different metabolites were identified in cancer species, and a few hundreds of them were found to be associated with prostate cancer.

## 7. Conclusions and Future Directions

Abnormal lipid metabolism has been associated with different pathologies, including cancer. There has been a growing number of studies that investigate the lipid metabolic profile and signaling pathways using the combination of different analytical platforms for quantitative and semi-quantitative analysis. MS coupling with chromatographic separations (e.g., GC, LC) or direct infusion mass spectrometry (DIMS) are methods extensively used in lipidomics research. Moreover, lipidomics can provide insights into cancer, namely BC, as it can help in the discovery of molecules used as disease biomarkers and in drug development. Additionally, one of the major challenges in lipidomics is to obtain comprehensive information about the lipidome. In order to accomplish more discoveries in the lipid research field, the development of new statistical tools that will be combined with the mass spectrometric methods is required to improve data processing, identification, and interpretation of critical biological pathways involved in BC development. Among the methods presented in this review, MALDI MSI combined with multivariate statistical analysis seems to be the most promising, and that could become routine in clinical laboratory and oncology. However, certain improvements in the sample preparation and instrument settings are required.

## Figures and Tables

**Table 1:** Fragments of phospholipids that can be identified in MALDI spectra by applying collision induced decay (CID). The fragments that are characteristic for an individual PL class are listed.

Class	Fragments, Positive Ion Mode	Fragments, Negative Ion Mode	References
PC	*m/z* 184, Δ59 (neutral loss of choline), Δ183 (loss of phosphocholine), loss of fatty acid (corresponding LPC)	*m/z* 168	[81]
SM	*m/z* 184, Δ59 (neutral loss of choline)	*m/z* 168	[81,82]
PE	*m/z* [M-H-141]+, loss of FA (corresponding LPE)	*m/z* 140	[81]
PI	*m/z* 417	*m/z* 241	[81]
PS	*m/z* [M-H-185]+	*m/z* [M-H-87]−	[81]
Fatty acyl ions	*m/z* 239 (C16:0), *m/z* 267 (C18:0), m/z 287 (C20:4)	*m/z* 255 (C16:0), *m/z* 283 (C18:0), *m/z* 303 (C20:4)	[81,82]

## Abbreviations

AFAI	air flow-assisted ionization
ANOVA	analysis of variance
AUC	area under the curve
BC	breast cancer
BMP	bis(monoacylglycero)phosphate
COX 1 and COX 2	cyclooxygenases 1 and 2
cPLA_2_	cytosolic PLA_2_
CV	cross validation
DAG	diacylglycerol
DAG	diacylglycerol
DCIS	breast ductal carcinoma in situ
DESI	desorption electrospray ionization
DIMS	direct infusion mass spectrometry
ESI	electrospray ionization
FA	fatty acid
FAK	focal adhesion kinase
FFA	free fatty acid
GC	gas chromatography
GC-MS	gas chromatography-mass spectrometry
GPCR	heterotrimeric G-protein coupled receptor
HILIC	hydrophilic interaction chromatography
IDC	breast invasive ductal carcinoma
iPLA_2_	calcium-independent
LC	liquid chromatography
LC–ESI/MS	liquid chromatography-electrospray ionization mass spectrometry
LC–PB–MS/MS	liquid chromatography tandem mass spectrometry
LPA	lysophosphatidic acid
LPCAT1	lysophosphatidylcholine acyltransferase 1
MALDI	matrix assisted laser desorption/ionization
MAPK/ERK	mitogen activated protein kinase/extracellular-signal-regulated kinase
MSI	mass spectrometry imaging
MUFA	monounsaturated fatty acid
NF-κB	light-chain-enhancer of activated B cells
NMR	nuclear magnetic resonance
NPLC	normal phase liquid chromatography
OPLS-DA	orthogonal partial least-squares discriminant analysis
PA	phosphatidic acid
PAF-AH	platelet-activating factor acetylhydrolase
PC	phosphatidyl choline
PCA	principal components analysis
PI3K	phosphatidylinositol-3-kinase
PIP_2_	phosphatidylinositol 4,5-bisphosphate
PKC	protein kinase C
PKCδ	protein kinase C delta type
PL	phospholipid
PLA	phospholipase A
PLC	phospholipase C
PLD	phospholipase D
PLS-DA	partial least squares discriminate analysis
PUFA	polyunsaturated fatty acid
RIA	radioimmunoassay
ROC	receiver operating characteristic curve
SATA	saturated fatty acid
sPLA_2_	phospholipase A_2_
TGs	triglycerides
TNF α	tumor necrosis factor α
TOFMS	time-of-flight mass spectrometry
UHPLC	ultra-high performance liquid chromatography
UHPSFC	ultra-high performance supercritical fluid chromatography
